# Part three: a randomized study to assess biomarker changes in cigarette smokers switched to Vuse Solo or Abstinence

**DOI:** 10.1038/s41598-022-25054-z

**Published:** 2022-11-30

**Authors:** Milly N. Kanobe, Bobbette A. Jones, Paul Nelson, Buddy G. Brown, Peter Chen, Patrudu Makena, Eckhardt Schmidt, John Darnell, John W. Caraway, G. L. Prasad, Brian Nordskog, Elaine K. Round

**Affiliations:** 1 RAI Services Company, 401 N. Main Street, Winston-Salem, NC 27101 USA; 2 Prasad Scientific Consulting LLC, 490 Friendship Place Ct, Lewisville, NC 27023 USA; 3 JTI Leaf Services (US) LLC, 202 Stinson Drive, Danville, VA 24540 USA

**Keywords:** Biomarkers, Diseases

## Abstract

Biomarkers of exposure (BoE) can help evaluate exposure to combustion-related, tobacco-specific toxicants after smokers switch from cigarettes to potentially less-harmful products like electronic nicotine delivery systems (ENDS). This paper reports data for one (Vuse Solo Original) of three products evaluated in a randomized, controlled, confinement study of BoE in smokers switched to ENDS. Subjects smoked their usual brand cigarette ad libitum for two days, then were randomized to one of three ENDS for a 7-day ad libitum use period, or to smoking abstinence. Thirteen BoE were assessed at baseline and Day 5, and percent change in mean values for each BoE was calculated. Biomarkers of potential harm (BoPH) linked to oxidative stress, platelet activation, and inflammation were also assessed. Levels decreased among subjects randomized to Vuse Solo versus Abstinence, respectively, for the following BoE: 42–96% versus 52–97% (non-nicotine constituents); 51% versus 55% (blood carboxyhemoglobin); and 29% versus 96% (nicotine exposure). Significant decreases were observed in three BoPH: leukotriene E4, 11-dehydro-thromboxane B2, and 2,3-dinor thromboxane B2 on Day 7 in the Vuse Solo and Abstinence groups. These findings show that ENDS use results in substantially reduced exposure to toxicants compared to smoking, which may lead to reduced biological effects.

## Introduction

It is well known that cigarette smoking is a leading cause of preventable premature death and disease, including cancer, cardiovascular disease, and chronic obstructive pulmonary disease (COPD)^1,2^. In 2012, the US Food and Drug Administration (FDA) established a list of harmful and potentially harmful constituents (HPHCs) in tobacco products. Embedded in this list, FDA categorized each HPHC as a carcinogen, respiratory toxicant, cardiovascular toxicant, reproductive or developmental toxicant, and/or addictive^3^. The type of tobacco product used, and the frequency, duration, and manner of use are associated with the level of exposure to HPHCs as well as disease risk^4^. Thus, a continuum of risk for different classes of tobacco- and nicotine-containing products is recognized, where the use of combustible tobacco products, such as cigarettes, is established as the most harmful form of tobacco use and the use of FDA approved nicotine products are considered one of the least harmful^5^. It must be noted, that to avoid continued exposure to all HPHCs and to reduce harm from smoking, complete cessation of smoking is the best option^6^.

Biomarkers of exposure (BoE) are used as measures of exposure to tobacco and tobacco smoke constituents in humans. Several clinical studies have previously confirmed that individuals who significantly reduce their daily cigarette consumption (> 50%) have somewhat lower levels of BoE relative to those who continue to smoke^7–10^. Data from the Population Assessment of Tobacco and Health study reveal that complete quitters have substantially reduced BoE^11^.

Electronic nicotine delivery systems (ENDS) fall between cigarettes and nicotine replacement therapies on the continuum of risk^4,12^. The aerosol generated during the use of ENDS has been characterized as containing lower levels of known toxicants compared to those found in cigarette smoke^13–15^, and significantly fewer toxic constituents than traditional cigarettes^16–21^. Emerging research has shown significantly lower levels of urinary carcinogen and toxicant metabolites in ENDS users compared to cigarette smokers^19^, as well as significant declines in biomarkers of tobacco exposure among cigarette smokers switched exclusively to ENDS^18,22,23^. For example, smokers who were completely switched to a Vuse ENDS product showed marked reductions in biomarkers of several representative HPHCs, including nicotine, tobacco specific nitrosamines (TSNAs), volatile organic compounds (VOCs), polycyclic aromatic hydrocarbons (PAHs) and aromatic amines. These reductions were similar in magnitude to those observed in individuals who completely abstained from use of any tobacco product^23^.

Validated functional predictive biomarkers, also known as surrogate biomarkers of complex smoking-related diseases such as lung cancer, COPD and cardiovascular disease, do not exist^24,25^. Alternatively, tobacco-related biomarkers of potential harm (BoPH) provide an interim assessment of biological effects resulting from tobacco product exposure that can lead to the development of disease. Such biomarkers can serve as intermediate indicators of disease risk from tobacco product exposure and can be measured in the context of short-term switching studies.

Chronic smoking is associated with elevated oxidative stress and inflammation, which are key drivers of smoking-induced pathophysiology^1^. Smoking-induced oxidative stress and inflammatory response are indicated by an increased synthesis of arachidonic acid (AA) and its metabolites^26–28^. A panel of AA-derived metabolites, including prostaglandins, isoprostanes, thromboxanes, and leukotrienes, has been demonstrated as a useful set of short-term BoPH in smokers who switch from smoking to abstinence or tobacco products lower on the risk continuum for as few as five days^29^.

This study assessed the effects of switching non-menthol smokers from combustible cigarettes (CC) to use of one of three Vuse ENDS products (Vuse Solo [G2], Vuse Vibe or Vuse Ciro) or Abstinence, at Day 5 of a seven-day in-clinic switch. For the purposes of this manuscript, data resulting from those subjects randomized to the Vuse Solo and Abstinence groups will be reported. Data for the other Vuse ENDS product groups will be reported in a later publication.

The primary endpoints in this study were validated BoE to constituents recommended for evaluation in ENDS e-liquids and aerosols as listed in the draft guidance for Premarket Tobacco Product Applications for ENDS published by the US FDA Center for Tobacco Products in May 2016^30^ and/or compounds identified by the FDA as HPHCs^3^. All of the tobacco-related BoE quantified in this study have been reviewed previously^31^. The secondary objectives of this study were to evaluate daily product use amounts and to assess urinary AA-derived metabolites as BoPH at Day 5 and Day 7 following an in-clinic switch from UB cigarettes to use of Vuse Solo or Abstinence. The Abstinence group was included in the study design to assess the maximum biomarker change possible when subjects experience the same environment and conditions as those who switch to an ENDS product but stop use of tobacco products completely.

Several of the AA metabolites are mediators of important physiological processes such as platelet activation and airway inflammation, and are known to be increased in chronic cigarette smokers, potentially contributing to increased cardiovascular risk and airway obstruction, respectively. Previous studies have reported rapid changes in a subset of AA metabolites upon smoking abstinence^32,33^. Results from this study build upon the existing body of research which suggests that exclusive use of ENDS products poses less individual health risk to tobacco product consumers than CC smoking.

This paper is the third in a three-part series reporting on three recent studies conducted to evaluate the abuse liability^34^, nicotine pharmacokinetics^35^, and biomarkers of Vuse Solo (current). Adverse event (AE) information for all three studies is collectively reported in this paper.

## Results

This randomized, controlled study was designed to assess BoE in smokers switched to one of three closed Vuse ENDS product groups or Abstinence during an in-clinic confinement. Only the data for subjects randomized to either the Vuse Solo or Abstinence groups will be reported here. No between-group comparisons were performed.

### Study population

Thirty-five subjects were randomized to the Vuse Solo group and 16 to the Abstinence group. Among these, 46 (90%) completed the study (Vuse Solo group n = 35, Abstinence group n = 11). Four subjects in the Abstinence group voluntarily withdrew from the study, and one was discontinued due to an AE. Subject demographics and baseline characteristics are summarized in Supplementary Table S1. The study population was similar between the two groups and was generally balanced between males (57.1%, 56.3%) and females (42.9%, 43.8%) for Vuse Solo and Abstinence, respectively. The subject population was predominantly white (74.3% [Vuse Solo]; 68.8% [Abstinence]) and non-Hispanic (91.4% [Vuse Solo]; 100.0% [Abstinence]), with a mean age of 41.2 years for Vuse Solo and 39.9 years for Abstinence. Subjects in both the Vuse Solo and Abstinence groups self-reported being exclusive CC smokers for an average of 27.3 and 24.4 years, respectively, with both groups reporting the average number of cigarettes smoked per day as 18.3. The overall level of cigarette dependence at baseline was moderate for both groups based on Fagerstrӧm Test for Nicotine Dependence scores (mean total score of 6.1 and 6.0 for Vuse Solo and Abstinence groups, respectively).

### Biomarkers of exposure in urine and blood

**Figure 1:**
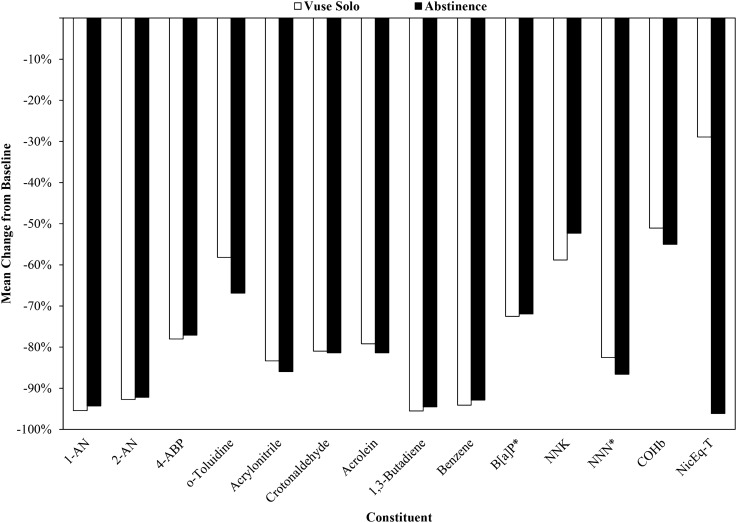
Mean percent change from baseline in constituent biomarkers of exposure in smokers switched to Vuse Solo or Abstinence for 5 days. 1-AN (1-aminonaphthaline; ng/24 h); 2-AN (2-aminonaphthalene; ng/24 h); 4-ABP (4-aminobiphenyl; ng/24 h); *o-Tol* (o-Toluidine; ng/24 h); Acrylonitrile (2-cyanoethyl mercapturic acid [CEMA]; µg/24 h); Crotonaldehyde (3-hydroxy-1-methylpropylmercapturic acid [HMPMA]; µg/24 h); Acrolein (3-hydroxypropyl mercapturic acid [HPMA]; µg/24 h); 1,3-Butadiene (Monohydroxybutenyl mercapturic acid [MHBMA]; µg/24 h); Benzene (S-phenyl mercapturic acid [SPMA]; µg/24 h); B[a]P (3-hydroxy-benzo[a]pyrene; pg/24 h); NNK: (Nicotine-derived nitrosamine ketone; 4-(methylnitrosamino)-1-(3-pyridyl)-1-butanol + glucuronides [Total NNAL]); NNN (N-nitrosonornicotine; N’-nitrosonornicotine + glucuronides [Total NNN]; ng/24 h); COHb: carboxyhemoglobin (%); NicEq-T: Nicotine ([Total Nicotine Equivalents]; ng/24 h); all changes from baseline to Day 5 were statistically significant, with the exception of NNN in the Abstinence group due to one extreme data value.

Thirteen BoE (12 urinary and one blood) were evaluated as primary endpoints in this study. Levels of all evaluated BoE in both study groups statistically significantly (*p* < 0.05) decreased five days after switching from UB cigarettes to use of Vuse Solo or Abstinence (Fig. 1; Supplementary Table S2). Aromatic amines decreased 58–95% and 66–94% in the Vuse Solo and Abstinence groups, respectively. For biomarkers of semi-volatile organics, reductions ranged from 79 to 96% in the Vuse Solo group and 81–95% in the Abstinence group. One polycyclic aromatic hydrocarbon was measured in this study, 3-OH-benzo[a]pyrene (3-OH-B[a]P). Although statistically significant (*p* = 0.0420, step-down Bonferroni-adjusted), only a 42% reduction in the biomarker of benzo[a]pyrene (B[*a*]P) was observed in the Vuse Solo group due to ~ 50% of subjects having baseline values below the lower limit of quantification (100 fg/mL; Supplementary Table S4). A statistically significant (*p* = 0.0015) reduction of 72% in the biomarker of benzo[a]pyrene (B[*a*]P) was observed in the Abstinence group. Statistically significant (*p* < 0.05) reductions were observed for tobacco-specific nitrosamines, with values ranging from 59–83% to 52–97% in the Vuse Solo and Abstinence groups, respectively. Additionally, a statistically significant (*p* < 0.0001) reduction of 83% was observed for total NNN in the Vuse Solo group versus a non-significant (*p* = 0.1131) reduction of 97% in the Abstinence group. When one extreme value was excluded from the Abstinence group Day -1 dataset, the reduction of total NNN was 87% and statistically significant (*p* = 0.0061).

Similar to reductions in urinary BoE, blood carboxyhemoglobin (COHb) levels were statistically significantly (*p* < 0.05) reduced 51% and 55% post-product switch (Day 5) for the Vuse Solo and Abstinence groups, respectively (Fig. 1; Supplementary Table S2).

### Total nicotine equivalents (NicEq-T)

Decreases in urinary NicEq-T (Unconjugated nicotine [Nic-U] and the 5 metabolites [Unconjugated cotinine, Unconjugated trans-3´-hydroxycotinine, Nicotine-N-glucuronide, Cotinine-N-glucuronide, and Trans-3´-hydroxycotinine-O-glucuronide] converted to molar Nic-U equivalents and summed [NicEq-T]) were statistically significant (*p* < 0.05) between baseline (Day-1) and Day 5, with reductions of 29% and 96% observed for the Vuse Solo and Abstinence groups, respectively (Fig. 1; Supplementary Table S2).

### Biomarkers of potential harm

Switching from smoking to either Vuse Solo or Abstinence resulted in decreases in AA-derived metabolites (Table 1). Smokers switched to Vuse Solo for seven days showed statistically significant decreases in leukotriene E4 (LTE4) of 28% on Day 5 and 34% on Day 7 (*p* < 0.001 for both days). Significant decreases were also observed for 11-dehydro-thromboxane B2 (11-dh-TXB2) on both Day 5 (23%, *p* < 0.001) and Day 7 (32%, *p* = 0.001), while 2,3-dinor thromboxane B2 (2,3-d-TBX2) decreased 23% (*p* = 0.061) and 51% (*p* = 0.001) on Days 5 and 7, respectively.

**Table 1:** Urinary levels of LTE4, 11-dh-TXB2, and 2,3-d-TXB2 in smokers switched to Vuse Solo or Abstinence for 5 and 7 days.

Group	Biomarker of potential Harm	Total mass (ng/24 h) ± SD			*p*-value from paired t-test		% change	
		Baseline (Day-1)	Day 5	Day 7	Baseline versus day 5	Baseline versus day 7	Baseline versus day 5 (%)	Baseline versus day 7 (%)
Vuse Solo (N = 35)	LTE4	138.3 ± 63.3	98.9 ± 52.5	91.8 ± 53.5	< 0.001	< 0.001	− 28	− 34
	11-dh-TXB2	614.9 ± 369.7	471.5 ± 242.1	417.4 ± 167.0	< 0.001	0.001	− 23	− 32
	2,3-d-TXB2	764.6 ± 540.6	585.2 ± 479.1	378.0 ± 305.4	0.061	0.001	− 23	− 51
Abstinence (N = 16)	LTE4	111.5 ± 58.0	58.2 ± 38.1	72.7 ± 57.4	0.001	0.032	− 48	− 35
	11-dh-TXB2	492.8 ± 200.7	388.2 ± 238.8	355.4 ± 217.2	0.121	0.017	− 21	− 28
	2,3-d-TXB2	937.1 ± 804.3	444.4 ± 393.7	625.0 ± 437.3	0.041	0.044	− 53	− 33

*LTE4* leukotriene E4, *11-dh-TXB2* 11-dehydro-thromboxane B2, *2,3-d-TXB2* 2,3-dinor thromboxane B2, *SD* standard deviation.

Smokers switched to Abstinence for seven days showed statistically significant decreases in LTE4 of 48% (*p* = 0.001) on Day 5 and 35% (*p* = 0.032) on Day 7. For 11-dh-TXB2, decreases of 21% (*p* = 0.121) and 28% (*p* = 0.017) were observed on Day 5 and Day 7, respectively; while 2,3-d-TXB2 decreased significantly by 53% (*p* = 0.041) and 33% (*p* = 0.044) on both Days 5 and 7, respectively.

### Daily E-liquid consumption

The mean amounts of Vuse Solo e-liquid consumed each day of the seven-day product switch period are presented in Table 2. These data show that Vuse Solo e-liquid consumption remained relatively constant on Days 1 and 2, increased over Days 3 through 5, with a slight decrease from maximum consumption on Days 6 and 7 (Table 2).

**Table 2:** Amount of Vuse Solo e-liquid used per day (g) over the 7-day study period among 35 subjects randomized to Vuse Solo group.

Time Point	n	Mean ± SD	Min, Max
Day 1	35	0.44 ± 0.28	0.02, 1.17
Day 2	35	0.44 ± 0.27	0.04, 1.18
Day 3	35	0.46 ± 0.25	0.07, 1.14
Day 4	35	0.50 ± 0.26	0.04, 1.15
Day 5	35	0.58 ± 0.29	0.06, 1.28
Day 6	35	0.55 ± 0.25	0.05, 1.35
Day 7	35	0.55 ± 0.27	0.02, 1.23

*N* total number of subjects randomized to Vuse Solo group, *n* number of subjects who used Vuse Solo on a given study day, *SD* standard deviation, *Min* minimum amount of Vuse Solo e-liquid used, *Max* maximum amount of Vuse Solo e-liquid used.

### Adverse events

Forty-nine adverse events (AEs) were reported, 36 AEs among 21 subjects in the Vuse Solo group and 13 AEs among 10 subjects in the Abstinence group. No serious AEs were reported. Eighteen (50%) of the AEs reported in the Vuse Solo group were determined by the Principal Investigator (PI) to be unlikely or not related to product use. A total of 10 AEs (27.8%) were deemed possibly related to Vuse Solo use and 8 AEs (22.2%) were deemed related. Most AEs were mild in intensity, and none were severe. The most common AEs reported in the Vuse Solo group included headache (11.4%), cough (5.7%), back pain (8.6%), dry throat (5.7%), toothache (5.7%), dyspepsia (5.7%) and musculoskeletal pain (5.7%). Those AEs determined by the PI to be possibly or definitely related to Vuse Solo use included headache (two subjects), dry throat, dyspepsia, chest discomfort, dyspnea, lip blister, and oropharyngeal pain (one subject each). The most common AEs reported in the Abstinence group were irritability (12.5%) and back pain (12.5%). These AEs occurred after randomization; therefore, none of the AEs were related to product use. No subject in the Vuse Solo group discontinued the study as a result of an AE. However, one subject in the Abstinence group was discontinued due to an AE, which the PI determined was related to nicotine withdrawal symptoms.

Across this study and the other two reported as part of this series, 53 total AEs were reported by 68 subjects using Vuse Solo^34,35^. Of these, the study PI determined 11 AEs were possibly related and 9 AEs were related to Vuse Solo use (Supplementary Table S3). All AEs causally related to Vuse Solo use were mild, and included headache, cough, back pain, oropharyngeal pain, musculoskeletal pain, nausea, dry mouth, dry throat, and gastroesophageal reflux (Supplementary Table S3).

## Discussion

This paper reports findings from a study that evaluated BoE and BoPH changes in non-menthol CC smokers after a short-term in-clinic switch to use of Vuse Solo or abstinence for seven days. Statistically significant (*p* < 0.05) decreases from baseline to Day 5 were observed in the majority of the urinary BoE assessed and blood COHb. Levels of all 12 urinary BoE evaluated were significantly (*p* < 0.05) reduced at Day 5 for the Vuse Solo group. Similar statistically significant (*p* < 0.05) reductions were observed in the Abstinence group, with the exception of total NNN. Although the mean percent reduction in 3-OH-B[a]P was statistically significant (*p* < 0.05) in both Vuse Solo and Abstinence groups, the reduction was less (42%) in the Vuse Solo group compared to that for the Abstinence group (72%). Extreme data values (data that were beyond the interval mean ± 6 times the standard deviation for each endpoint) were observed for both 3-OH-B[a]P in the Vuse Solo group and total NNN in the Abstinence group.

Statistical analyses for 3-OH-B[a]P and total NNN were performed with and without the extreme data values identified. When the subjects with extreme data values for 3-OH-B[a]P were excluded from the analysis (n = 2 of 35), the reductions in this BoE remained statistically significant at *p* < 0.05, although the percent change from baseline increased to 73%, similar in magnitude to the reduction observed in the Abstinence group (72%). There were a few highly concentrated urine samples in the range of 1000 fg/mL for 3-OH-B[a]P. Since all batch criteria (calibration, quality controls and incurred sample reanalysis) were fulfilled at the analytical laboratory and the concentrations were still within the calibration range (50.3–3221.3 fg/mL), no re-analyses of the samples were performed. In addition, even though there was a possibility that exposure to B[a]P came from sources other than smoking (mainly occupational exposure), the observed levels were not extraordinarily high, so smoking could still remain the main source. The analytical laboratory used a highly sensitive, high throughput method for the quantification of 3-OH-B[a]P^36^. Additionally, the reported baseline values of 3-OH-B[a]P for about 50% of subjects in the Vuse Solo group were below the lower limit of quantification. Therefore, the percent changes observed were mostly dependent on the respective 24-h urine volume collected. Therefore, the observed extreme data values for the 3-OH-B[a]P could be accounted for by random variability as opposed to analytical variability. Comparable results of low baseline levels of 3-OH-B[a]P in smokers switched short-term to an ENDS product were also reported in a previous study by^23^. For total NNN, when the subject with the extreme data value in the Abstinence group (n = 1 of 11) was excluded from the analysis, the 97% reduction in total NNN changed from non-significant (*p* = 0.1131) to a statistically significant reduction of 87% (*p* = 0.0061). The excretion of NNN in urine can be highly biased due to the formation of NNN by nitrosation of nornicotine under acidic conditions, both endogenously and exogenously. After screening the data set, only one exceptionally high value (261.6 pg/mL) was found in one subject (261.6 pg/mL; the 261.6 pg/mL value also falls under the category “Extreme data values). The high NNN value from this subject was found in the Day-1 sample and correlated *very well with levels of other typical smoking markers measured (high NicEq-T, 3-OH-B[a]P, and CEMA levels).* After the confinement period (Day 5), all biomarkers showed a clear decline in Vuse Solo group, which was in line with the Abstinence group. Considering that this subject was a smoker (baseline), all precursors for endogenous NNN formation were present. Thus, the elevated levels could be explained by the mechanism as outlined in the systematic evaluation of endogenous urinary NNN formation^37^.

In general, the reductions in total NNAL were lower (59% and 52% in Vuse Solo and Abstinence groups, respectively) compared to other urinary biomarkers assessed, and the mean percent reductions were similar between both groups. The elimination half-life of NNAL is 10–45 days^7,38,39^, and this study assessed changes in NNAL five days after product switch. Therefore, the reductions observed do not represent the extent of the reductions that would be observed over a longer period of switching.

The reduction in levels of all urinary BoE were similar to values reported previously for an earlier version of Vuse Solo (G1)^23^. In that study, Round et al. reported on 153 cigarette smokers who switched from UB cigarettes to Vuse Solo or nicotine gum for five days in a clinical confinement setting. The authors reported mean total reductions in BoE of 30–99% for all the groups evaluated, and the extent of reduction for the Vuse Solo groups was similar to that of the nicotine gum groups.

Results of this study are also consistent with results of switching to other ENDS products reported elsewhere. Jay and colleagues^22^ evaluated changes in BoE in adult cigarette smokers who either completely switched from CC to one of four pod-based ENDS products or abstinence, or who continued smoking CC. They evaluated nine biomarkers of exposure (NNN, NNAL, 3-HPMA, MHBMA, SPMA, HMPMA, CEMA, COHb and 1-OHP), of which eight (all except 1-OHP) were assessed in this study. They reported statistically significant reductions in toxicant exposure of up to 85.0% and 85.3% in smokers who switched to the ENDS product and abstinence, respectively. Hecht et al.^19^ reported statistically significantly lower levels of 1-OHP, total NNAL, 3-HPMA, 2-HPMA, HMPHM, and SMPA among ENDS users compared to cigarette smokers. Other researchers have also reported lower levels of urinary BoE among smokers who switched short-term to use of ENDS^17^.

The mean percent reduction in blood COHb observed in this study’s Vuse Solo group was similar to the mean percent reduction observed in the Abstinence group (51% vs. 55%, respectively; Fig. 1). Jay et al.^22^ reported 69–74% COHb reduction in the ENDS groups compared to 69% reduction in the Abstinence group. The results in the current study are also comparable to the earlier reported findings by Round and colleagues^23^.

Reductions in NicEq-T in the Vuse Solo group, although statistically significant (*p* < 0.05), were smaller than the reductions observed for other urinary biomarkers in this group. NicEq-T decreased significantly by 29% in the Vuse Solo group versus the statistically significant 96% reduction in the Abstinence group. These findings demonstrate the expected biomarker reductions with use of a nicotine-containing product, which indicates that the subjects in the Vuse Solo group remained exposed to nicotine levels closer to baseline UB smoking relative to those in the Abstinence group. These results support the concept that smokers who switch to Vuse Solo will use the product in such a way to provide them with levels of nicotine near those of smoking, while reducing their exposure to tobacco-related toxicants found in cigarette smoke. Further evidence to demonstrate that nicotine uptake occurs with Vuse Solo use, but not to the same extent as when smoking a CC, are presented in Campbell et al.^34^. In their study, Campbell et al. reported on the abuse liability of Vuse Solo, which showed that mean peak nicotine uptake from 10-min ad libitum use of Vuse Solo was 61% less (*p* < 0.05) than nicotine uptake from smoking one UB cigarette.

As a part of continued qualification of BoPH that rapidly respond to changes in smoking status, this study also evaluated select AA- metabolites. A key finding was that switching to exclusive use of Vuse Solo or Abstinence resulted in reductions of LTE4, 11-dh-TXB2, and 2,3-d-TXB2. While several different BoPH exist, our previous work has shown that these three BoPH are elevated in smokers, relative to non-smokers and users of non-combustible tobacco, and decline rapidly in smoking cessation^29,40^. These findings align with studies which demonstrate that urinary LTE4 and 2,3-d-TXB2 levels return to baseline within two weeks of tobacco product abstention^41^. This similar trend toward reduction of urinary LTE4 levels was observed in smokers switched to either Vuse Solo or smoking abstinence.

Thromboxane A2 (TXA2) is a short-lived eicosanoid derived from cyclooxygenase-1 (COX-1)-dependent metabolism of arachidonic acid. TXA2 is very rapidly metabolized to Thromboxane B2 (TXB2) and further metabolized to 11-dehydro TXB2 and 2, 3-dinor TXB2. It is reported that increased levels of LTE4 are linked to airway inflammation; whereas increased levels of 11-dh-TXB2 and 2,3-d-TXB2, are reflective of platelet activation that leads to atherosclerosis and cardiovascular disease^42–45^. Thus, the decline in LTE4, 11-dh-TXB2, and 2,3-d-TXB2 levels in smokers who switch to noncombustible tobacco products suggests a potential for reduction in airway inflammation and platelet activation, which are the two key drivers of smoking-induced pathophysiology. These short-term improvements in the BoPH are encouraging and indicate beneficial biological responses to switching smokers to exclusive Vuse ENDS usage. However, the normalization of BoPH levels, along with marked declines in BoE, would need to be sustained over the long-term in smokers who switch to exclusive use of Vuse ENDS products for any eventual improvements in physiological changes.

While the data presented herein reflect the effects in cigarette smokers who completely switch to Vuse Solo use, there are limitations to the study design. First, to ensure that subjects used only the ENDS product or abstained from smoking, the study was conducted in a confined setting, which reduces the variability of environmental toxicant exposure due to the controlled study environment. However, a confined setting intentionally limits options for product use. While these data are indicative of exposure changes if cigarette smokers completely switch to Vuse Solo, in a “real-life” environment, where cigarette smokers are able to freely choose the tobacco products they use, other patterns of product use are possible. Dual use and poly use of tobacco products, which may occur in an ambulatory setting, could result in different toxicant exposures.

Secondly, this study did not include a group of subjects who continued to smoke, which would have allowed an assessment of the difference between BoE levels from continued smoking and switching to Vuse Solo or abstinence under the same study conditions. Some have reported that urinary BoE significantly (*p* < 0.001) increased in the continued smoking groups in a confinement setting^22,46^. Other studies that investigated BoE in a randomized controlled design have reported increased exposure to toxicants in the continued smoking group^10,47,48^. Given these results, a similar continued smoking group in the current study may have impacted study results by possibly increasing or decreasing the differences from baseline. As such, the biomarker results presented here may represent the worst-case scenario for biomarker reduction given the results from prior research^22,46^.

The results from this study demonstrate that short-term switching from smoking to use of Vuse Solo leads to decreased exposure to tobacco smoke constituents. The reductions in toxicant exposure observed among subjects switched to Vuse Solo were comparable in magnitude to the reductions observed in subjects who completely abstained from smoking, suggesting that the reduction in exposure for the Vuse Solo group was similar to that of the Abstinence group for the biomarkers assessed.

Assessment of abuse liability is another important regulatory requirement of premarket evaluation of novel tobacco products in determining whether they are appropriate for the protection of the public health (APPH)^49^. Evaluation of the abuse liability of Vuse Solo (G1) has revealed that the original and menthol variants exhibit significantly lower abuse liability compared to cigarettes, but closer to nicotine gum^50,51^. In agreement with these findings, independent studies also have revealed that different ENDS products have lower abuse liability than cigarettes, and are effective in relieving smoking withdrawal and craving symptoms with no reported side effects^52,53^. The findings presented in this study, along with the results reported in the abuse liability studies^50,51^, suggest that use of Vuse Solo may aid in product adoption by current smokers. Moreover, in October 2021, the FDA CTP granted marketing authorization for Vuse Solo (G1 and G2) with tobacco-flavored e-liquids. The results of the current study using Vuse Solo (G2), together with biomarker results on Vuse Solo (G1)^23^ formed part of the basis for FDA’s decision to deem these products as APPH^54^.

With the well-known risks of cigarette smoking, ENDS such as Vuse Solo can serve a beneficial purpose for public health if both the abuse potential and exposure to toxicants are reduced compared to CC. Taken together, the results presented in this series of studies demonstrate that Vuse Solo has a nicotine pharmacokinetic profile and an overall product liking profile that may provide an acceptable alternative to CC for smokers. Additionally, switching to Vuse Solo from CC reduces exposure to smoking-related toxicants. Collectively, these results provide further support for the role of closed system ENDS products in general, and Vuse Solo specifically, in tobacco harm reduction.

## Methods

### Study design

This randomized, controlled, switching, open-label, parallel cohort confinement study (ClinicalTrials.gov registration number; date: NCT03170674; 31/05/2017) was conducted between April and September 2017 at two clinical research sites (Overland Park, KS and Minneapolis, MN), contracted by Altasciences Clinical Research as the contract research organization (CRO). The study was reviewed and approved by MidLands Independent Review Board (Overland Park, KS, USA) and was conducted in accordance with applicable sections of the United States Code of Federal Regulations (21 CFR Parts 50, 54, and 56), and the International Conference on Harmonisation (ICH) Good Clinical Practice (ICH-E6, 1996), which is consistent with the Declaration of Helsinki. All subjects provided written informed consent prior to any study procedures being performed and were free to withdraw from the study at any time and for any reason. Subjects were compensated for their time and participation.

This study was designed to primarily assess BoE and BoPH in smokers switched short-term to Vuse Solo ENDS product, herein referred to as Vuse Solo (G2) or Vuse Solo. Although we evaluated the study endpoints for three distinct products (Vuse Solo, Vuse Vibe and Vuse Ciro), the purpose was not to compare among products but rather to evaluate each product independently. In addition, the results from this study were intended to support regulatory applications for each product independently. The approach to evaluate all three products in the same clinical study was to optimize resources at the clinical sites, and to meet study/regulatory timelines for each of the products. The results from the other two products (Vuse Vibe and Vuse Ciro) are being considered for publication elsewhere.

Generally healthy males and females ages 21–60 years who self-reported smoking ≥ 10 combustible, filtered, non-menthol cigarettes per day for at least 6 months prior to screening, confirmed by an expired carbon monoxide (ECO) level of ≥ 12 parts per million and a positive urine cotinine test (≥ 200 ng/ml), were included in the study. The subjects were required to be willing to switch from using their UB cigarettes to use of one of three Vuse ENDS products or to remain abstinent from smoking through the end of the study, depending on their randomization assignment. Other eligibility criteria were also assessed by the PI; however, women who were pregnant, breastfeeding, or intended to become pregnant during the study were excluded from participation.

Potential subjects completed a pre-screening telephone interview and also attended a screening visit at the clinical research site within 30 days before study entry. Eligible subjects were enrolled into the study on Day-2 and stayed at the clinical site for a 10-day confinement period. The subjects were allowed to smoke their UB cigarettes ad libitum, between the hours of 0700 and 2300 for the first two days (Days-2 and -1), during which baseline measurements were taken. On Day-2, the level of dependence on cigarettes was assessed with the Fagerström Test for Nicotine Dependence, on which a score of 0–2 indicates low dependence and a score of 8–10 indicates high dependence^55^.

On Day 1, subjects were randomized to either a Vuse Solo ENDS IP group or the Abstinence group. A computer software program was used by the statistician at the CRO to generate the randomization sequences. A total of 35 and 15 subjects were randomized in the Vuse Solo and the Abstinence groups, respectively. There were 10 blocks total for Vuse Solo (7 blocks, sequence code B) and for the Abstinence (3 blocks, sequence code D) groups. The study was unblinded by necessity due to the very different visual appearances of the study products. Upon randomization, the subjects in the Vuse ENDS groups were allowed to use their assigned ENDS IP ad libitum between the hours of 0700 and 2300. The randomized subjects were supervised and monitored by the clinical site staff to ensure study product usage and protocol compliance.

Sampling for urinary BoE was performed two times by collecting 24-h urine samples beginning on the mornings of Day-1 and Day 5 through the mornings of Days 1 and 6, respectively. Blood samples for measurement of COHb concentrations in whole blood were collected once a day (at approximately 12 h post start of product use) on Days-1, 1, 3, and 5.

Daily ENDS IP usage was controlled by the clinical staff and restricted after 2300 h on Days 1 through 7. The amount of IP consumed per subject per day was measured as the total mass of e-liquid used per day. This was done by weighing the cartridges before and after use and allowing the subjects to use the same cartridge until entirely depleted.

Additional urinary samples were collected on study Days-1, 5, and 7 for the evaluation of BoPH. To determine whether AA metabolism was altered following short-term switching, a panel of nine urinary AA-derived metabolites were measured in both the Vuse Solo (n = 35) and Abstinence (n = 11) groups after five and seven days of switching from UB smoking.

Safety evaluations were assessed by monitoring AEs, physical examinations (including oral examinations), electrocardiograms, clinical laboratory tests (chemistry, hematology, virology, and urinalysis), pregnancy screening, and vital sign measurements. An AE was defined as any untoward medical occurrence or condition experienced by a subject from randomization (on Day 1) until completion of the study, whether or not considered related to the use of IP by the PI. Any AEs with a start time prior to randomization was recorded as part of the subject’s medical history. The severity of AEs was categorized as mild (of little concern to the subject and/or of no clinical significance), moderate (discomfort enough to cause interference with or change in usual activities), or severe (incapacitating or unable to work or subject in many or all usual activities; is of concern to the subject and/or poses substantial risk to the subject’s health or well-being). In all cases, the PI made a determination of the relationship of the AE to the IP using a four-category system (not related, unlikely related, possibly related, or related).

The data presented in this manuscript were collected from baseline (Day-1) through the morning of Day 8. The subjects were discharged from the study on Day 8 after completing all the End of Study Assessments.

### Study product

Vuse Solo, the ENDS product that is the subject of this manuscript, comprises a rechargeable Vuse Solo power unit (battery capacity: ≥ 270 mAh; wattage: 3.00 W) and a closed cartridge containing 0.5 mL of Original (tobacco-flavored) e-liquid, a mix of propylene glycol (PG), glycerin (PG:glycerin ratio of 21:79), water, and flavoring ingredients. The e-liquid included nicotine at 4.8% nicotine by weight (57.4 mg/mL) and contained nicotine salts. The results of this current paper pertain only to the Vuse Solo (G2 cartridge design), which is substantially similar to the Vuse Solo (G1 cartridge design) product that was used in prior studies^23,50^, with slight differences in materials and electronics configuration. All products used in the study were commercially available in the USA at the time the study was conducted.

### Biomarker analyses

All urinary and blood tobacco constituents were analyzed at Analytisch-Biolgisches Forschungslabor (ABF) GmbH, in Munich or Planegg, Germany. All sample handling, analysis and data reporting activities were performed in accordance with applicable principles of Good Laboratory Practice (GLP) as set forth in the Code of Federal Regulations, Title 11, Part 58, and in accordance with current ABF Standard Operating Procedures. ABF has established and validated analytical methods for the biomarkers included in this study. Analytical method validation was performed according to FDA Guidelines for Industry (FDA, 2001). All analytical methods used are accredited by ISO 17025.

All methods have been published previously^36,56–61^ except those for COHb, which are briefly described below. A summary of all methods, lower limits of quantification, and limits of detection are provided in Supplementary Table S4 online.

Carboxyhemoglobin was measured by mixing 100 μL blood samples with 1.25 mL internal standard solution (^13^CO-saturated blood diluted with water 1:500) and 200 μL water in a headspace vial. A total of 200 μL potassium ferricyanide (200 g/L in water) was added. Release of carbon monoxide was achieved by stirring the vial for 10 s at 750 rpm. After incubation for 30 min at 55 °C, the headspace was injected on to the gas chromatography–mass spectrometry system that included an Agilent gas chromatograph (6890N) coupled to an Agilent mass spectrometer (MS5975). Chromatographic separation was performed using a Restek Rt-Msieve 5A PLOT column (30 m × 0.32 mm and thickness 30 μm) at 45 °C. Helium was used as carrier gas (flow 1.9 mL/min). The injector was set to 40 °C with a 3:1 split ratio. The mass spectrometer was run in the selected ion mode with electron ionization.

For analysis of BoPH, LC–MS/MS was used to measure a panel of urinary AA metabolites including PGF2α, 8-i-PGF2α, 2,3-d-8-i-PGF2α, t-PGDM, t-PGEM, 2,3-d-TXB2, 11-dh-TXB2, LTE4, and 12(S)-HETE at ABF GmbH, München, Germany^29,32,62^.

### Statistical analysis

Among tobacco combustion biomarkers, B[*a*]P is notably more variable between individuals than the other BoE previously tested^10,23^. Therefore, the sample size was based on the number of subjects needed to detect a statistically significant reduction in this BoE from baseline to Day 5. We estimated that if 31 subjects in the Vuse Solo group completed the study, we would achieve 80% power with a Bonferroni-adjusted significance level (α = 0.0038, calculated as 0.05/13 for the number of comparison tests) to detect at least a 65% reduction in overall B[*a*]P concentration at an overall significance level of 0.05. Therefore, approximately 35 subjects were planned to be enrolled and randomized in the Vuse Solo group. No formal sample size calculations were performed for the Abstinence group.

The primary endpoints were assessed with 13 statistical comparison tests (including 12 urinary and one blood biomarker comparisons). For urinary BoE, raw concentrations (mass/mL) were reported by the bioanalytical laboratory. Total urinary amounts excreted per day (total mass/day) were determined by multiplying mass/mL x total urine output volume in mL over 24 h. The statistical analysis was performed on total mass/day for all urine BoE. Total nicotine equivalents (NicEq-T) was treated as a secondary endpoint. For the blood BoE (COHb), the bioanalytical laboratory reported percent (%) carbon monoxide. Descriptive statistics (mean and standard deviation [SD]) were calculated for the 12 urinary BoE and blood COHb for baseline during cigarette smoking (Day-1) and post-product switch (Day 5). Changes in these primary BoE from Day-1 to Day 5 were assessed for statistical significance using a one-sided paired t-test, except for NicEq-T where statistical significance was assessed using a two-sided paired t-test. Percent (%) change from Baseline (Day-1) to post-product switch (Day 5) was also calculated for both the urinary and blood BoE.

To account for multiplicity and preserve the overall type I error rate of 0.05, each test on the primary endpoints was adjusted using the adaptive step-down Bonferroni method implemented in SAS version 9.4 (SAS Institute, Cary NC, USA)^56^. No multiplicity adjustments were made for the secondary endpoint. Biomarker data were screened for potential extreme data values. Extreme data values were defined as data that were beyond the mean ± 6σ for each endpoint according to internal SOP. If any extreme data values were observed, analyses were repeated with and without the extreme data values. No comparisons between the Vuse Solo and the Abstinence groups were performed.

The BoPH levels are presented as total mass excreted in 24 h (ng/24 h). Descriptive statistics were calculated for baseline (Day-1) and for Day 5 and Day 7 post-product switch to either Vuse Solo or Abstinence. To compare the differences between baseline and post-switching in each group, a paired t-test was conducted to determine statistical significance (*p* < 0.05). Percent (%) change from Baseline (Day -1) to post-product switch (Days 5 and 7) was also calculated for BoPH. No adjustments were made for multiple comparisons. JMP 10 (SAS Institute, Cary, NC, USA)^63^ was used for data analysis.

Descriptive statistics of demographics, baseline characteristics, and Vuse Solo use (e-liquid consumption) are summarized by group, and time point where appropriate. All enrolled subjects were included in these summarizations, as well as the data reported for AEs.

## Supplementary Information

Supplementary Information.

## Data Availability

The applicable data generated or analyzed during this study are included in this manuscript (and its supplementary Tables). Additional datasets generated and/or analyzed during the study are available from the corresponding author on reasonable request if deemed necessary.
