# Supplementary material for: Part three: a randomized study to assess biomarker changes in cigarette smokers switched to Vuse Solo or Abstinence
Source: Sci Rep. 2022 Nov 30;12:20658. doi: 10.1038/s41598-022-25054-z (PMC9712618; doi:10.1038/s41598-022-25054-z)
Supplement: Supplementary file 1 — Supplementary Information. [file 41598_2022_25054_MOESM1_ESM.docx]

**Part Three: A Randomized Study to Assess Biomarker Changes in Cigarette Smokers Switched to Vuse Solo or Abstinence**

Milly N. Kanobe^1,*^, Bobbette A. Jones^2^, Paul Nelson^2^, Buddy G. Brown^1^, Peter Chen^1^, Patrudu Makena^1^, Eckhardt Schmidt^1^, John Darnell^1^, John W. Caraway^1^, G.L. Prasad^2,3^, Brian Nordskog^4^, and Elaine K. Round^1^

^1^RAI Services Company, 401 N. Main Street, Winston-Salem, NC 27101, USA

^2^Retired employees of RAI Services Company

^3^ Prasad Scientific Consulting LLC, 490 Friendship Place Ct, Lewisville, NC 27023, USA

^4^JTI Leaf Services (US) LLC, 202 Stinson Drive, Danville, VA 24540, USA

***Corresponding Author:**

Milly Kanobe, PhD

RAI Services Company

401 N. Main Street

Winston-Salem, NC, 27101, USA

336-741-0934

[kanobem@rjrt.com](mailto:kanobem@rjrt.com)

# Supplementary Table S1. Subject demographics and baseline characteristics

| **Characteristic** | **Vuse Solo (n = 35)** | **Abstinence (n = 16)** |
| --- | --- | --- |
| Age, years | 41.2±11.2 | 39.9±8.0 |
| Weight, kg | 81.2±16.3 | 83.2±19.4 |
| Height, cm | 172.5±8.0 | 172.2±10.4 |
| BMI, kg/m^2^ | 27.4±6.0 | 28.0±5.4 |
| Sex |  |  |
| Male | 20 (57.1%) | 9 (56.3%) |
| Female | 15 (42.9%) | 7 (43.8%) |
| Ethnicity |  |  |
| Hispanic/Latino | 2 (5.7%) | 0 |
| Not Hispanic/Latino | 32 (91.4%) | 16 (100.0%) |
| Not reported | 1 (2.9%) | 0 |
| Race |  |  |
| White | 26 (74.3%) | 11 (68.8%) |
| Black/African American | 7 (20.0%) | 4 (25.0%) |
| Native Hawaiian/Other Pacific Islander | 0 | 0 |
| Asian | 0 | 0 |
| American Indian/Alaska Native | 1 (2.9%) | 0 |
| Multiple | 0 | 1 (6.3%) |
| Not Reported | 1 (2.9%) | 0 |
| Smoking |  |  |
| Number of years smoked | 27.3±10.9 | 24.4±11.2 |
| Number of cigarettes smoked per day | 18.3±6.9 | 18.3±7.1 |
| FTND | 6.1±1.6 | 6.0±1.3 |

Data are mean±SD or number (%).

Abbreviations: BMI = body-mass index; FTND = Fagerstrӧm Test for Nicotine Dependence

# Supplementary Table S2. Changes in biomarkers of exposure

|  | |  | **Vuse Solo  (n=35)** | | |  | **Abstinence (n=16)** | | | | |  |
| --- | --- | --- | --- | --- | --- | --- | --- | --- | --- | --- | --- | --- |
| **Urinary Biomarker (units)** | **Tobacco Constituent** | | **Day –1**  **(Mean±SD)** | **Day 5**  **(Mean±SD)** | **% change** | **p-value** | **Day –1**  **(Mean±SD)** | **Day 5**  **(Mean±SD)** | | **% change** | | **p-value** |
| ***Aromatic* *Amines*** |  | |  |  |  |  |  |  | |  | |  |
| 1-Aminonaphthaline  (ng/24 hours) | 1-aminonaphthalene | | 110.77±43.30 | 5.08±2.81 | –95.41% | <0.0001 | 93.27±55.58 | 5.34±3.55 | | –94.28% | | 0.0006 |
| 2-Aminonaphthalene  (ng/24 hours) | 2-aminonaphthalene | | 27.37±12.01 | 2.00±1.22 | –92.70% | <0.0001 | 27.55±14.39 | 2.15±1.01 | | –92.20% | | 0.0003 |
| 4-Aminobiphenyl  (ng/24 hours) | 4-aminobiphenyl | | 21.53±7.35 | 4.73±3.01 | –78.02% | <0.0001 | 19.45±9.32 | 4.46±2.45 | | –77.07% | | 0.0003 |
| *o*-Toluidine (ng/24 hours) | *o*-Toluidine | | 203.39±87.49 | 85.09±59.23 | –58.17% | <0.0001 | 204.36±125.18 | 67.70±25.90 | | –66.70% | | 0.0055 |
| ***Semi-volatile Organics*** |  | |  |  |  |  |  |  | |  | |  |
| CEMA (µg/24 hours) | Acrylonitrile | | 257.27±100.46 | 42.90±22.00 | –83.33% | <0.0001 | 245.87±125.18 | 34.57±22.44 | | –85.94% | | 0.0002 |
| HMPMA (µg/24 hours) | Crotonaldehyde | | 515.22±213.14 | 98.01±47.80 | –80.98% | <0.0001 | 521±64 | 97.18±53.90 | | –81.37% | | 0.0006 |
| HPMA (µg/24 hours) | Acrolein | | 1.62±0.60 | 0.34±0.16 | –79.19% | <0.0001 | 1.63±1.00 | 0.30±0.11 | | –81.37% | | 0.0020 |
| MHBMA (µg/24 hours) | 1,3-butadiene | | 2.91±2.02 | 0.13±0.09 | –95.52% | <0.0001 | 2.47±1.80 | 0.14±0.05 | | –94.51% | | 0.0022 |
| SPMA (µg/24 hours) | Benzene | | 5.34±3.11 | 0.31±0.21 | –94.10% | <0.0001 | 4.70±3.15 | 0.34±0.21 | | –92.86% | | 0.0014 |
| ***Polycyclic Aromatic Hydrocarbon*** |  | |  |  |  |  |  |  | |  | |  |
| 3-OH-B[*a*]P (pg/24 hours) | Benzo[a]pyrene | | 240.68±323.64 | 139.32±437.19 | –42.11% | 0.0420 | 218.70±217.70 | 61.38±43.61 | | –71.94% | | 0.0233 |
| 3-OH-B[*a*]P (pg/24 hours)** | Benzo[a]pyrene | | 240.68±323.64 | 66.16±62.79 | –72.51% | 0.0015 | 218.70±217.70 | 61.38±43.61 | | –71.94% | | 0.0117 |
| ***TSNAs*** |  | |  |  |  |  |  |  | |  | |  |
| Total NNAL (ng/24 hours) | 4-(methylnitrosamino)-1-(3-pyridyl)-butanol) | | 668.71±318.51 | 275.41±165.32 | –58.81% | <0.0001 | 600.59±404.24 | 286.51±166.15 | | –52.30% | | 0.0062 |
| Total NNN (ng/24 hours) | N’-nitrosonornicotine | | 11.82±11.88 | 2.06±1.40 | –82.54% | <0.0001 | 63.45±165.86 | 2.11±0.80 | | –96.68% | | 0.1131 |
| Total NNN (ng/24 hours)** | N’-nitrosonornicotine | | 11.82±11.88 | 2.06±1.40 | –82.54% | <0.0001 | 15.71±13.20 | 2.11±0.80 | | –86.60% | | 0.0061 |
| ***Nicotine*** |  | |  |  |  |  |  | |  | |  |  |
| Total nicotine equivalents  (mg/24 hours)* | Nicotine | | 17.05±6.44 | 12.12±7.75 | –28.91% | 0.0010 | 16.79±8.80 | | 0.65±1.10 | | –96.12% | <0.0001 |
| **Blood Biomarker (units)** | |  |  |  |  |  |  |  | |  | |  |
| Carboxyhemoglobin (%) | Carbon monoxide | | 11.52±2.84 | 5.64±1.94 | –51.04% | <0.0001 | 11.73±3.95 | 5.28±1.10 | | –55.01% | | 0.0005 |

Abbreviations: CEMA = 2-cyanoethyl mercapturic acid, HMPMA = 3-hydroxy-1-methylpropyl-mercapturic acid, HPMA 3-hydroxypropyl mercapturic acid, MHBMA = monohydroxybutyl mercapturic acid, SPMA = S-phenyl mercapturic acid, 3-OH-B[*a*]P = 3-hydroxy-benzo[*a*]pyrene, TSNAs = tobacco specific nitrosamines,

NNAL = 4-(methylnitrosamino)-1-(3-pyridyl)-1-butanol, NNN = *N*’-nitrosonornicotine, SD = standard deviation.

* 24-hour urine total nicotine equivalents

** Biomarker results excluding extreme value(s)

# Supplementary Table S3. Adverse Events reported with relationship to Vuse Solo across studies

|  | Campbell et al. | Hong et al. | Present Study |
| --- | --- | --- | --- |
| # Adverse Events | 8 | 9^a^ | 36 |
| # of Subjects | 38 | 9 | 21 |
| # Possibly Related to product | 0 | 1 | 10 |
| # Related to product | 0 | 1 | 8 |

^a^ Denotes only those adverse events that were captured during portions of the study when subjects used Vuse Solo

# Supplementary Table S4. Tobacco constituent and analytic methods

| **Urinary Biomarker** | **Tobacco Constituent** | **Abbreviation** | **Matrix** | **Method** | | **LOD** | **LLOQ** |
| --- | --- | --- | --- | --- | --- | --- | --- |
| 1-aminonaphthalene | 1-aminonaphthalene | 1-AN | Urine | GC-MS | | 0.6 ng/L | 1.8 ng/L |
| 2-aminonaphthalene | 2-aminonaphthalene | 2-AN | Urine | GC-MS | | 0.57 ng/L | 1.7 ng/L |
| 4-aminobiphenyl | 4-aminobiphenyl | 4-ABP | Urine | GC-MS | | 0.51 ng/L | 1.5 ng/L |
| *o*-toluidine | *o*-Toluidine | *o-Tol* | Urine | GC-MS | | 0.83 ng/L | 10 ng/L |
| 2-cyanoethyl mercapturic acid | Acrylonitrile | CEMA | Urine | LC-MS/MS | | 0.08 ng/mL | 0.25 ng/mL |
| 3-hydroxy-1-methylpropyl-mercapturic acid | Crotonaldehyde | HMPMA | Urine | LC-MS/MS | | 0.49 ng/mL | 5 ng/mL |
| 3-hydroxypropyl mercapturic acid | Acrolein | HPMA | Urine | LC-MS/MS | | 12.58 ng/mL | 25 ng/mL |
| Monohydroxybutyl mercapturic acid | 1,3-butadiene | MHBMA | Urine | LC-MS/MS | | 0.029 ng/mL | 0.129 ng/mL |
| S-phenyl mercapturic acid | Benzene | SPMA | Urine | LC-MS/MS | | 5.0 pg/mL | 0.02 ng/mL |
| 3-hydroxy-benzo[*a*]pyrene | Benzo[a]pyrene | 3-OH-B[*a*]P | Urine | LC-MS/MS | | 33.3 fg/mL | 100 fg/mL |
| 4-(methylnitrosamino)-1-(3-pyridyl)-1-butanol) + glucuronides | 4-(methylnitrosamino)-1-(3-pyridyl)-butanol) | Total NNAL | Urine | LC-MS/MS | | 2 pg/mL | 5 pg/mL |
| N’-nitrosonornicotine + glucuronides | N’-nitrosonornicotine | Total NNN | Urine | LC-MS/MS | | 0.8 pg/mL | 2 pg/mL |
| Total nicotine equivalents | | NicEq-T* | Urine | Calculated |  |  |  |
| Nicotine | Unconjugated Nicotine | Nic-U | Urine | LC-MS/MS | | 0.5 ng/L | 1.5 ng/L |
| Cotinine | Unconjugated Cotinine | Cot-U | Urine | LC-MS/MS | | 0.17 ng/L | 0.51 ng/L |
| Cotinine | Unconjugated trans-3’-hydroxycotinine | OH-Cot | Urine | LC-MS/MS | | 0.21 ng/L | 0.63 ng/L |
| Nicotine | Nicotine-*N*-glucuronide | Nic-Gluc | Urine | LC-MS/MS | | 0.17 ng/L | 0.51 ng/L |
| Cotinine | Cotinine-*N*-glucuronide | Cot-Gluc | Urine | LC-MS/MS | | 0.33 ng/L | 0.99 ng/L |
| Metabolite of trans-3'-hydroxycotinine | *Trans-*3’-hydroxycotinine-O-glucuonide | OH-Cot-Gluc | Urine | LC-MS/MS | | 0.75 ng/L | 2.25 ng/L |
| **Blood Biomarker** |  |  |  |  | |  |  |
| Carboxyhemoglobin | Carbon monoxide | COHb | Whole Blood | HS-GC-MS | | 0.40% | 0.75% |

Abbreviations: GC = gas chromatography, MS = mass spectrometry, LC = liquid chromatography, HS = headspace, LOD = limit of detection, LLOQ = lower limit of quantification.
*NicEq-T = Nic-U and the 5 metabolites converted to molar Nic-U equivalents and summed.
